# Pathological Physiology, or Clinical, Experimental, and Microscopical Researches on Inflammation, Tuberculization, Tumours, the Formation of Callus, &c.

**Published:** 1846-07

**Authors:** 


### ART. II.

*Physiologie Pathologique, ou Recherches cliniques, expérimentales, et microscopiques sur l'Inflammation, la Tuberculization, les Tumeurs, la formation du Cal, &c.* Par H. LEBERT, M.D. Accompagné d'un Atlas de vingt-deux planches gravées. Deux Tomes.—*Paris,* 1845.

*Pathological Physiology, or Clinical, Experimental, and Microscopical Researches on Inflammation, Tuberculization, Tumours, the formation of Callus, &c.* By H. LEBERT, M.D. With twenty-two engraved plates. 2 Vols.—*Paris,* 1845. 8vo, pp. 554 and 515.

THE volumes we are about to notice contain the first attempt yet made in the French language to present a general view of the minute nature and constitution (as discoverable with the microscope) of the processes and products of disease. The work is entitled to a welcome in France, where a very remarkable state of ignorance concerning the advances made of late years in this country and in Germany in the department in question prevails. The amount of favour with which it may claim to be received *here* must depend upon the amount of novelty in fact, inference, or doctrine it contains—upon the degree of critical acumen it exhibits in separating the mountains of dross from the sprinkling of gold scattered through the recent literature of pathology, and upon the fairness and conscientiousness with which the labours of the author's predecessors are employed and acknowledged. It is, at least, upon grounds such as these that our award shall be made.

The author divides his account of inflammation into two chapters. In the first appears an inquiry into the general phenomena of inflammations, those common (more or less invariably) to that state as it occurs in all the tissues and organs; in the second we are made acquainted with the peculiarities of the disease in certain textures.

The general history of inflammation commences with that of the state of the capillaries and blood in the parts actually implicated in the morbid process. As the greater part, indeed almost the entire, of the description here given not only contains no novelty, but bears upon facts which, though announced with considerable pomp and an ingenuous tone of self-sufficient originality, are as familiar to all minute observers of inflammation as redness is to the surgeon among its clinical phenomena, we shall only notice a few statements scattered through these prolix pages. The author holds that in the first stage of inflammation the caliber of the capillaries is diminished, and the rapidity of the circulation increased; he should have known that this has been questioned altogether by some very sagacious microscopists, and that, at least, it is *certainly* not a constant occurrence. He estimates the possible amount of diminution at one third of the total width of the vessel. This attempt at metrical precision appears to us to render inaccuracy more inaccurate, rather than anything else.

M. Lebert takes credit to himself for the announcement that the generation of new vessels in inflammation is "not exclusively confined to the organization of the products of exudation," but occurs amid the tissues themselves affected with the hyperæmic changes. A precious *discovery* this! which happens to have been taught by one of the very earliest minute observers of the inflammatory process, Kaltenbrunner.* It is true that Kaltenbrunner supposed that extravasated blood channeled canals for itself in the tissues amid which it escaped, and that M. Lebert does not admit this mode of production; but the main *fact*, put forward as novel, is as old as the study of inflammation. M. Lebert introduces with considerable flourish of trumpets his explanation of the mode in which new vessels are formed: "He has specially engaged in this investigation, entering as it did into the plan of his pathological researches, and of his study of development in general." (p. 15.) Scarcely will it be believed that after this morsel of egotistical puffing this author should have neither investigated in any novel manner any old facts, nor traced out any new ones, and that he leaves the three notions, which have long contended for supremacy in the minds of pathologists, (namely, that new tubes are produced by the elongation or looping process from old vessels—that they are merely vessels which pre-existed, but which being too narrow to receive red blood, have, through the inflammatory process, acquired sufficient width to admit this—and, lastly, that they are actual new formations generated independently of the pre-existing circulation,)—that he leaves these three notions precisely as he found them, unproved and unsettled. Nay, more, he is either deplorably ignorant of researches made in this country, in Germany,

* See Cyclopædia of Medicine, art. Inflammation, p. 713.

and in Italy, on the subject; or he conceals his acquaintance with them for motives which we do not pretend to understand.

There is nothing new here on the phenomena of hyperinosis; but it is as well to make it known to the readers of this Journal that the "great fact" of hyperinosis, increased proportion of fibrine, had been pointed out by Stannius and Denis in 1838. The fame of M. Andral, *fils,* has thrown into shade too profound the merits of the original observers of the facts which he has confirmed and generalized.

The "compound-inflammation corpuscles" of Gluge receive a description of considerable length and much pretension at the hands of M. Lebert. He adds nothing to our knowledge of their nature, and there is something ludicrous in the solemnity with which he declines to admit the error of Gluge (the error of, however, an accurate and truly original observer), that the corpuscle in question is formed of aggregated nuclei of stagnating red corpuscles. M. Lebert believes the constituent granules rather of fatty nature than composed of fibrine or albumen. This is announced in the guise of an original opinion. It is well known, however, that such was the notion held and taught by Valentin and Ascherson. The German observers gave ingenious and most plausible, if not absolutely convincing, reasons for the views they advocated. M. Lebert contents himself with a magisterial statement of his opinion; he goes back instead of advancing. And he finishes off this lame and yet conceited description by referring to his figures of these corpuscles—figures which are, beyond all comparison, the very worst representations of the real bodies we have ever chanced to see.

The few next pages contain some similar announcements of the discoveries of others, (without any reference to the original observers,) which we conceive it is well to notice. Speaking of the fibrinous matter of common plastic pleural exudation, M. Lebert observes, "the fibrine becomes thus more and more condensed, and the appearance of *fibroid* stratification gradually changes into real fibrous tissue, but not through the intervention of cells and fusiform corpuscles; it is rather effected by condensation of the effused materials." Passing over the elegancies of diction of this sentence, its hybrid word, and its reference to an *appearance changing into a tissue,* let the reader note the honesty of the man who, in announcing such a theory, omits all mention of the name of Mr. Gulliver. In the next page we find M. Lebert assuming a quiet tone of superiority on the strength of the discovery, that in various states of morbid texture, described as "cartilaginous transformations," no really cartilaginous structure exists. Bless the man's eagle ken! Why Müller (to whose 'Archiv' M. Lebert is a contributor, be it observed,) showed the non-cartilaginous nature of many products, (to which the term had formerly been applied,) seven years ago; and the fact is as familiarly known to all persons acquainted with the progress of minute morbid anatomy—("practical surgeons" of course continue, and will continue for the next half century, to speak of "loose *cartilages*" in the joints, &c.; but it is to be presumed M. Lebert does not write for *them*,)—as one and one make two. Similarly is the statement made that in the so-called "osseous transformation" of false membrane no bone is to be found. True it certainly is, that the amorphous mineral matter, the patches of saline substance, found often in various other morbid productions as well as in pseudo-membrane, were set down as actual "ossifications" by certain morbid anatomists before the introduction of the microscope; and true it also is, we believe, that some of these morbid anatomists having once "said it, stick to it." But it is quite as true that the novelty of M. Lebert has been for years matter well known to all accurate observers. To all appearance M. Lebert fancies himself addressing an ignorant and a dishonest audience. What empty *fanfaronade* is this that meets our eye in his preface concerning his having chosen the French language as the medium for issuing to the world his production?—"It was for him a matter of conscience and of deep satisfaction to write in the tongue of that land (although not his maternal language) where he had received such support and sympathy!" &c. &c. No, M. Lebert! There may be a truer explanation found for your eschewing your mother-tongue, and taking refuge in the convenient (under the circumstances) language of France. Could you have written in the language of Valentin, of Henle, of Güterbock, of Gluge, of Ascherson, and of many other honest, honorable, and high-minded Germans, of whose patient labours your work seems too often an appropriation? No, you could not; and so you bethought you of your patrons, those excellent Parisians, who, with a few distinguished exceptions, never at any time see much beyond their own noses in such matters, and who (experience doubtless taught you this) would be ready, once you became "one of them," to fight your battles for priority, originality, and all forms and varieties of what, in their inflated bombast, they call "GLORY." We tell you, M. Lebert, "glory" is a fine thing, but common honesty is a better thing. And trouble us not with the ready reply of literary thieves belonging to *your* section of the fraternity: "You do me injustice—see how original I am—look at my scores of cases—my jottings down of measurements of cells—my post-mortem examinations, and my clinical records." Trouble us not with this, we say, for we know its value; we know, we have traced, the way in which the thing is done. Are you a very original man if you take in hand, for example, Abercrombie on the 'Brain,' get thoroughly up the details of symptoms and morbid changes of some particular cerebral disease, and, thus crammed, make your way into a ward where some example of the sort is to be found, and from memory make up a "case," with profound "original" remarks at its tail concerning the disease in question—all of which remarks are to be found in essence in the standard volume that has instructed you? Hardly, we should think. And yet this is the most lenient way of viewing the course you have pursued, and which is pursued by many by whom temporary notoriety is taken in barter for honour.

M. Lebert having found existing descriptions of the proper pus-corpuscle "much less accurate than the deep importance of the subject requires," has "long made this body the subject of attentive observation." The prolonged and attentive observation has unfortunately led to the detection of no single point of novelty in the anatomy or pathology of the corpuscle; though the account is got up in a style to deceive many readers into a belief that much of the matter before them is original. Talking, for instance, of the action of acetic acid on the corpuscle, he observes as follows: "Several authors have contended that the nuclei of these corpuscles originally formed a single nucleus only, which split up into several under the action of the acid. We object to this mode of viewing the point, from the facts that the presence of several nuclei may be recognized without the action of the acid, and that we have never seen any appearance of intermediate degrees of the splitting up." Now, would not any human being, previously unacquainted with the subject, fancy that the merit of pointing out the error of the notion that the nucleus is invariably single in the natural state, and while uninfluenced by chemical reagents, belonged to M. Lebert? Yet the truth is, for years past, the fact, as put forward by him, has been known to all observers, and was *first* (as we believe) stated in print by Vogel in his work on Suppuration—a work, be it observed, with which M. Lebert is, on his own admission, acquainted. It may be advisable to add that the discovery that a double, treble, or quadruple nucleus might be seen in pus, to which no chemical agent had been added, was first made on pus that had stood for some time. The observation was consequently open to the objection that free acid might have been generated in the fluid, and acted, of course, as acid artificially added. Even this objection (of which M. Lebert appears, or desires to appear, ignorant) is without force. We, and doubtless many other persons, have frequently seen (with a power of 400 diameters even) a double or a treble nucleus in alkaline or neutral pus immediately on its removal from the body. On the whole, M. Lebert's description of the pus-corpuscle is greatly inferior both in accuracy and fulness to those of Henle and Vogel, though the former of these two persons was the originator of the error just referred to respecting the nucleus.

M. Lebert describes, as a constituent of pus, a corpuscle which he regards as a variety of the proper pus-corpuscle, and to which he gives the name of *pyoid.* "They are of spherical shape and composed of two elements; of a somewhat transparent substance, rather of solid consistence than liquid, and of molecular granules, varying in number from four to ten and upwards, irregularly scattered through their substance. They have no nucleus; and acetic acid, while it renders them somewhat more transparent, does not change them. They are larger and more spherical than the globules of tubercle, smaller and more granular in their substance than the white corpuscles of the blood, from which they differ in another essential character—their yellowish tint." We copy this description in order that our readers may inquire into its correctness. We have no acquaintance ourselves with a corpuscle of the kind, as a uniform or even habitual constituent of pus.

Pus contains more water than does the serum of healthy blood, or even blood in the state of inflammation. The sum of fibrine and of albumen is greater in pus than in the serum of inflammatory blood; as is also the quantity of fatty matter. The latter, as has long been known, abounds in some specimens of pus to such a degree as to render the fluid inflammable. Cholesteric fat, according to the recent numerous and most elaborate analyses of Bibra, chiefly accumulates—a fact confirmative of the statement of MM. Becquerel and Rodier, that the quantity of cholesterine in the blood constantly undergoes increase in inflammation. The quantity of ash proportionally left by the two fluids varies; there is sometimes more, sometimes less, in pus than in blood. The salts in the two fluids do not much differ. Hence the principal chemical difference is that the quantity of albumino-fibrinous substances is greater in the blood (the red corpuscles being included in the analysis), and the blood contains iron. Both these differences depend on the fact that in the process of pus formation the globules remain in the vessels, and that the fluid elements of the blood only transude through the vascular walls. A homœopathic dose of iron has, it is true, been sometimes found in pus, but this has arisen from accidental rupture of vessels, and consequent escape of red corpuscles. By the way, these chemical facts, and their aid in the demolition of that thrice-ridiculous notion, taught originally by Gendrin, and espoused by Donné, that the corpuscles of pus are transformed red blood-corpuscles. Doubtless the notion in question had been sufficiently well demolished by direct observation; but it is as well to point out the bearing of chemical evidence against its reception.

M. Lebert has endeavoured to trace the phenomena of pus-formation upon blistered surfaces, after the manner of Henle, Wood, Güterbock, and Vogel—to whose labours no reference is made. He has not been able to substantiate the affirmation of some authors that the nucleus is first formed, and the involucrum subsequently deposited around it. He fancies he has always observed that the pus-corpuscle, like many other cells, is formed with *all* its elements from the first—that these elements are at first excessively small, and that the ulterior change of the corpuscle consists in the increase of size, and in the clearer separation of the various elements which compose it.

M. Lebert is of opinion that there is no such thing as a special mucus-corpuscle, and that natural mucus, free from accidental admixture with substances foreign to itself, contains none of those corpuscles which have been described as belonging to it, and as very strongly similar in character to pus-corpuscles. When, he observes, the mucus examined was really healthy, young epithelial cells, or the large nuclei of old epithelial cells, were taken for special corpuscles, to which the name of mucus-corpuscle was assigned. Or, when the mucus examined was in a morbid state, these pus-corpuscles were mistaken for a novel variety of cell. We believe that this is a correct view of the question, which has certainly given rise to very contradictory statements both in this country and in Germany. But it is not to M. Lebert that the first establishment of the fact is due. The valuable observations of Vogel (in his work on Suppuration) contain the elements for the proper decision of the point; his experiments on the rapid production of pus-corpuscles, instead of epithelial cells, from the mucous membrane of the male urethra, under the slight irritation produced by the presence of a bougie, made the conclusion, which M. Lebert announces as perfectly novel, the only one tenable. Those experiments have also completely anticipated the results obtained by M. Lebert concerning the astonishing rapidity with which true pus-corpuscles are thrown off by mucous membranes. Vogel, too, goes into another and most interesting question, namely, to what extent can the formation of such corpuscles be considered to constitute true suppuration, while no proper fluid (no *liquor puris*) is at the same time produced, or at least can be shown to be produced. Here, as in many other places where M. Lebert puts himself forward as belonging to the vanguard, he is, in truth, bringing up the rear rather lazily.

In describing, (1) "the incomplete cure of suppurating wounds, and,

(2) cicatrization by granulation," M. Lebert attempts to introduce a modified version of the doctrine taught by various observers of the phenomena of cicatrization. The whole of the "novelty" here lies in a nutshell. The cell-structure of plastic exudation-matter, formed either on surfaces or in the interstices of organs, which in these positions forms the material of indurations, certain so-called hypertrophies, &c., and which is the product of chronic inflammation, M. Lebert describes under the name of fibro-plastic tissue. He describes the cells as going through the well-known series of changes from the spherical globule to the actual fibre. Now these corpuscles with their series of changes have certainly, as M. Lebert states, been by some writers described as the proper elements of granulations, and the means whereby perfect cicatrization is accomplished. To this doctrine he demurs, on the ground that the structure in question occurs under circumstances altogether opposed to the occurrence of perfect cicatrization, while this process is really effected on a very different plan. This plan may be made comprehensible according to M. Lebert by the following description: "Between the vessels of granulations," (the vessels are described in a loose manner, with not a tithe of the precision of Pauli and Miescher,) "lies a yellowish substance, appearing homogeneous and finely granular, when examined with a common lens, or with a glass of weak power; but if a stronger power be employed, the observer ascertains that this elastic semi-transparent tissue, this fibrinous gelatine is formed of a substance in which but few real fibres and few fusiform bodies are perceptible, but rather a stratification of fibrinous appearance, analogous in aspect to coagulated fibrine. Throughout this fibriform stroma lie round, or elongated, or deformed globules, measuring 0·1 millimeter in diameter, containing from two to four small nuclei, which are rendered more visible by the addition of acetic acid; these are deformed pus-globules in course of disintegration and solution. In a word, this intervascular substance is an organized pyo-blastema becoming transformed into coagulated fibro-albuminous gelatine, and holding imprisoned dissolving pus-globules." (Vol. i, p. 81.) If this last sentence be not the quintessence of absurdity, we know not what it is. Coagulated fibro-albuminous gelatine, which said most curious gelatine is an organized pyo-blastema! As cicatrization advances, the number of vascular loops diminish, as does that of the globules of pus: these at last cease to be distinguishable, "being decomposed into molecular granules; the *coagulated gelatine* itself becomes paler, and real fibres, delicate and distinctly defined, of unequal width (from ·002 to ·003 of a millimeter) constitute the chief part of the mass; they do not form fasciculi." Now, except in respect of the absurd language employed by the author, (we might indeed say the actually incorrect language,) there is absolutely nothing in all this. But now for the contents of his nutshell. "These fibres rather *appear* to be the final result of coagulation and condensation, than that of a transformation of cells and fusiform corpuscles." Now we are certainly as far from denying that this proposition would, if proved, be one of deep importance, as we are far from contesting the importance of the cell-theory itself; but we ask where is the evidence of its exactness in the details we have given, and these we pledge ourselves contain the essence of all that the author says upon the subject. Observe that he ventures no further than to affirm that the phenomena "appear"

to be of the kind he would fain have them, though the pompous exordium to the discussion on cicatrization justified us in expecting a thorough demonstration of some novel doctrine. And further, even if M. Lebert had proved his point, *his* would not be the merit of originality, as it is well known that the suggestion that fibres form independently of cells fell first from Mr. Gulliver. And, lastly, what evidence have we in the coarse descriptions, and still coarser figures of M. Lebert, that (admitting *argumenti gratiâ* that the fibres in question were not evolved from perfectly-formed cells) those fibres were not, as Henle contends is in some instances the fact, produced from nuclei arranged in rows and modified in character? To Henle's doctrine M. Lebert makes not even a passing allusion.

The *fibro-plastic* elements, as M. Lebert terms them, or simple induration matter, as others would less ambitiously entitle them, form "the basis of certain tumours, which we have often seen in the mammary gland, where they were taken for encephaloid; and we congratulate the surgeons who committed this error, as they will entertain the belief that they have radically cured cancer of the breast by operation." (p. 80.) To congratulate a man on his committing an error seems to us a very unaccountable mode of proceeding.

M. Lebert comes to the terminations of inflammation, ulceration and gangrene. He joins in the wake of those who maintain these two conditions to be very closely connected, on the ground that both acknowledge as their immediate cause the obliteration of a certain number of vessels, which are not replaced by others of new formation. The parts thus deprived of nutritious fluid "fall into detritus," that is, they ulcerate; or they "become detached in fragments of variable size," that is, they sphacelate. Now, there is little doubt (the notion has for some time been generally admitted,) that the connexion here pointed out between these two processes really exists; but, in the determination of this point, though an obvious step has been made, the difficulty of the theory of ulceration is by no means removed. It is perfectly clear that there is something besides obliteration of vessels to be discovered; for, if this were the whole morbid state existing, why should visible and tangible results, so widely differing as ulceration and gangrene, be both produced?

M. Lebert remarks on the treatment of inflammation. He examines the question of the utility of venesection and other modes of bloodletting, and leaves the question exactly as he found it, that is, undecided. He appears, indeed, to be but imperfectly acquainted with the method of seriously investigating a point in therapeutics, and is beguiled by the fancy that vague generalization from loose recollections may be permitted to assume the significance of sound inferences from lengthened and recorded experience. The following passage will supply justification of our mode of judging M. Lebert's claim to distinction as a guide in therapeutics, while (this is our motive for extracting it) it suggests for examination a question of very considerable interest:

"Having practised in a mountainous region, where many of the inhabitants, far removed from medical practitioners, are in the habit of leaving the treatment of diseases the most serious to the sole care of Nature, I have observed that persons labouring under inflammatory affections, especially pleurisy and pneumonia (frequent in this country), for which medical advice had been had from the first,

4299

and continued through the course of the disease,—I have observed, I say, that these persons generally recovered, and that, although they remained ill the length of time required by the course of the disease, they scarcely ever became the subjects of relapse or return of the disease during the subsequent years. Those patients, on the contrary, who had been allowed to remain without any kind of medical interference, often recovered, and sometimes sufficiently rapidly and completely; but, in general, the mortality was much greater among them, though *not* in proportion to the want of care that had been their lot. And the fact which struck me most forcibly was, that they were much more subject to return of the disease; and I have seen non-tuberculous persons, who declared that they had had three or four attacks of pleurisy in the course of a few years—a fact I have subsequently been enabled to verify myself." (vol. i, p. 96.)

We were not aware, until we read this passage (wherein the inference is forced upon us) that it is a very rare circumstance for persons to have three or four attacks of pleurisy in the course of a few years, even though they may have been bled, blistered, cupped, leeched, purged, nauseated, and the rest of it, during the first attack of the series. But, passing over this, we cannot avoid observing on the astonishing coolness with which this rural practitioner gives out a proposition of such deep importance as that we have just laid before the reader. Much as he is evidently disposed to overrate the value of his doings, we cannot conceive him so egotistical as to believe that a point so important as this should be considered to be established by his simple assertion; the most favorable view of the matter we can take is, that the vast bearings of the question mooted have altogether escaped his penetration.

Having passed in review the general phenomena of inflammation, M. Lebert turns to the consideration of inflammatory changes in various organs and textures. The numerous observations placed on record in this part of the volume are useful in some points, but, upon the whole, there is the usual deficiency of reference to the labours of others, and the same self-sufficient tone of a discoverer assumed here as elsewhere. In the description of "albuminous nephritis," we find the following statement concerning the "microscopical molecular composition of the renal granulations in Bright's disease:"

"I have found in them no other elements than very numerous molecular granules, varying in diameter from ·2 to ·25 of a millimeter, agglomerated in masses of some size. Frequently a certain number of these granules form into groups and eventually become surrounded with a membrane of investment,—a process analogous to the formation of the large globules of the yelk. These granular globules of ·15 to ·25 of a millimeter in diameter exhibit molecular movement in their interior; they sometimes exist in very notable quantity in the diseased kidneys. I have not succeeded in discovering them in the inside of the capillary vessels, notwithstanding all my efforts; and I believe that they are not found until after the escape of their elements by exosmosis through the walls of the capillaries. I have also found in the granulations fat-cells and fat-granules, sometimes even in tolerably large proportion." (vol. i, p. 147.)

We turn with M. Lebert to the anatomy of tubercle. The invariable elements of this substance he considers to be—1, a great quantity of perfectly round molecular granules; 2, a hyaline material uniting these granules, and the following element; 3, peculiar globules. These globules the author conceives to be the characteristic and essential element of tubercle; we must consequently place before the reader the chief points of the description given of them:

"The form of the globule of tubercle is rarely perfectly round . . . . . their somewhat angular outline depending probably on their close juxtaposition . . . . . they are of clear yellow colour, and appear blackish under a powerful glass. Their interior is irregular and of unequal consistence, which gives it a spotted appearance independently of the granules it contains. We have never been able to discover true nuclei in these corpuscles, although they sometimes present in their interior the irregular appearance of a vacuity resembling a nucleus. . . . . . The contained granules, disseminated irregularly through the substance of tuberculous globules, cannot be regarded as nuclei; they are, in fact, merely molecular granules, scarcely reaching, and never exceeding, a diameter of ·0025 of a millimeter. They vary in number from three to five, or ten and upwards; are not regularly placed in the interior of the globule, and not all visible at once on the same plane. . . . . . The diameter of the *round* tuberculous globule varies between ·005 and and ·0075 of a millimeter; the *oval* kind are of the mean length of ·0075 of a millimeter, by ·005 or ·006 in breadth. Their diameter increases when the process of softening commences." (vol. i, p. 353.)

Water is said not to affect these corpuscles of tubercle; acetic acid renders them more transparent without much altering them otherwise; above all, it discloses no nucleus in their interior. The latter statement stands in complete opposition to the description given by Henle.

Persons of much distinction as pathologists have, as M. Lebert observes, looked upon tuberculization as a modification of suppuration; and, besides, the existence of tuberculous matter in the interior of cancerous tumours has been frequently spoken of as a condition occurring in the dead body. The utter error of the first notion has been thoroughly proved by clinical observation; M. Lebert adds no fact to the evidence already existing on the subject in respect of microscopical anatomy, except his "tuberculous globule," as above described, be admitted among the acknowledged elements of tubercle: if so admitted, its presence or absence would of course constitute a point of testimony for or against the existence of tuberculization. With respect to the occurrence of tuberculous matter in cancerous tumours, much erroneous statement has gone abroad, and M. Lebert is right in exposing it; but, in exposing it, he is much less original than he supposes. Dr. Walshe, in his recent work on Cancer, has in several places made reference to the error in question, and (p. 83) even attempts, from the examination of two specimens of encephaloid, to refer the assumption of the tuberculous aspect by cancerous substance to the circumstance of the latter being associated with unusually large quantities of fat. Besides this, it was long since (as Dr. Walshe states) taught by Dr. Hodgkin, that encephaloid, having accidentally acquired a yellow tint, bears some resemblance to tuberculous matter.

Observing upon the well-known circumstance, that pus and softened tubercle are often found mixed together in particles examined under the microscope, M. Lebert insists, "*in contradiction,*" he says, "*to the generally admitted opinion,*" upon the fact that the pus does not come from the tubercle itself, but that it derives its origin from the circumjacent textures. This unquestionably takes us by surprise. As a matter of history in pathological anatomy we certainly have heard of the notion that tubercle was "a tissue, possessed of vessels, &c.," and as such "secreted" the pus wherewith it was found mingled; but that any one of the smallest authority in this country has (since the error of Laennec was exploded) held such a doctrine, we altogether deny, nor have we heard of its being professed either in France or in Germany.

In commenting on the fact that black pigmentary matter is sometimes found in considerable quantity around tubercles, M. Lebert observes that this occurs "especially in individuals *placed in circumstances particularly apt for the production of pigment,* as, for example, charcoal-men, who *introduce in breathing particles of carbon into the lungs in quantities,*—which substance has been analytically shown by M. Lecanu to be contained in those organs." (p. 371.) We confess we have much difficulty in understanding what the author's meaning is in this sentence,—the only one apparent on the surface is too ridiculous to have been entertained by him.

Here follows a section on the chemical properties of tubercle. The existing knowledge on the subject is put together in a very intelligible form. The fact recently established by M. Boudet, that there is much analogy in composition between the ash of tubercle and calcareous concretions forming in tubercles of the lungs or bronchial glands, is one of extreme interest.

M. Lebert confirms with the microscope the conclusion, established by clinical investigation by Laennec and Louis, that the gray granulation is a first stage of yellow tubercle:

"The invariable microscopical structure of gray granulations is a mixture of fibres, of a grayish coloured hyaline substance and the proper corpuscles of tubercle. When the gray granulation is seated in the lungs, the *fibres* are constituted by the *areolæ* [a curious inadvertence of language] of the elastic cellular fibres of the pulmonary tissue. If, on the contrary, these gray semi-transparent tubercles be seated in the subserous cellular tissue of a serous membrane, such as the peritoneum or the pia mater, the fibres are long, delicate, parallel to each other, and fasciculated. The proper elements of tubercle are always, however, to be discovered. From the absence or presence of the latter it is that we are enabled to determine whether certain granulations are tuberculous, or simply fibrous productions, which often remain as the last trace of pre-existing inflammation."

This, if true, is a very important point,—but we confess that all our experience leads us to doubt the *practicability* of the proposed mode of establishing the distinction in question.

M. Lebert has satisfied himself that fatty degeneration of the liver is by no means peculiar to phthisis, but occurs in a variety of chronic diseases of the alimentary canal. As respects the manner in which the fat is deposited in the liver, he has commonly found it in the form of fat vesicles, sometimes secreted in such quantity that the cells of the liver had altogether disappeared. But "the fat is very often deposited also in the interior of the cells of the liver, which proves that these cells are capable of imbibition and of undergoing various changes by means of endosmosis." We do not believe for our parts that the fact requires any such mode of explanation. No reference is made to the valuable observations of Mr. Bowman—observations, we happen to know, transferred to the French journals.

M. Lebert examines the question of the relationship of tuberculous and scrofulous disease—a question certainly of deep practical interest, and one which has been, from time to time, and is even at the present day, variously determined. He is of opinion that the affections, regarded by the majority of practitioners as scrofulous, may be divided into the three following classes:—1. Chronic inflammations, in which no specific character is really discoverable on careful inquiry, and which are not in point of fact of scrofulous nature. 2. Scrofulous diseases, properly so called. 3. Tuberculous diseases, which, although having a strong analogy to scrofula, should be separated therefrom, as well on clinical grounds as on the evidence of morbid anatomy. In respect to the latter point, he admits that the blood of scrofulous persons and that of tuberculous persons must be very closely related in character; but there is this difference, that tuberculous diseases generate a special substance, namely, tuberculous matter, characterized by peculiar globules, whereas the pus of scrofula does not, as far as M. Lebert's repeated examinations go, contain any special element distinguishing it from other morbid productions. A different doctrine has been taught concerning this latter point; it has been said that there is a distinct microscopical difference in the ingredients of scrofulous and healthy pus—the difference, or rather the material of the difference, has even been figured. We have not examined the point sufficiently to justify us in assuming the office of judge. We consider it right, however, to observe that the whole of M. Lebert's argument really turns upon his tuberculous globule and its non-existence in scrofulous pus. Now, of the complete accuracy of his narrative concerning that globule we feel particularly prone to doubt.

M. Lebert applies himself in his second volume to the consideration of tumours, and commences by dividing them into two classes, homœomorphous and heteromorphous,—a mode of classification which, though not very peculiarly novel, as the words merely take the place of the analogous and heterologous of Laennec and many of his followers, is put forward with the claims of an original thought. Cancer represents his heteromorphous class, because "the characteristic microscopical element of that product is not found in the natural state, either as a permanent or transitory element." Thus, then, he assumes there is a fixed character in a certain microscopical element, distinguishing cancerous from all other productions. Excellent well does this look on paper; but what says experience of it? Let us hear what Müller, a not insignificant spokesman in such a cause, declares: "The most innocent growths do not differ in their *minute elements*, nor in their origin, from carcinoma." M. Lebert continues: "as regards the distinctive characters of carcinoma, which have *hitherto* [!!] been given, such as its tendency to relapse, to invade adjoining textures, to become constitutional, to pass into the state of destructive ulceration, &c. &c., we recognize the value of these characters, especially when combined; but, on the other hand, we by no means discover in them characters exclusively proper to cancer." One would fancy that Müller had never written his book on Tumours, and, above all, that M. Lebert (though he quotes it frequently) had a most convenient faculty of forgetting its contents when seized with the ambition of appearing vastly original. There is no point on which Müller dwells more strongly than the inefficiency of these very characters as decisive of the cancerous nature of a tumour.

There is a good deal of novelty in M. Lebert's description of epithelial tumours, and we shall dwell more fully upon this chapter than upon any of his previous contributions. Epithelial tumours may be surrounded with a membranous investment, which is "either fibro-cellular, or wholly composed of globules." But epithelial tumours are also met with destitute of any special investment, and constituted under these circumstances by real hypertrophy of the epithelium: such, for example, are the nature and composition of opaque staphyloma.

> "The principal element of these tumours is composed of membranous layers of tesselated epithelium, closely juxtaposed, in the globules of which nuclei are generally very distinctly visible; vessels may exist in them in very small quantity. These stratified tumours are commonly of a dull white colour, sometimes of a yellowish tint; sometimes they are considerably vascular, and they then are of a reddish colour, while numerous vascular arborizations and sanguineous infiltration, caused by minute capillary effusions, are discoverable under the microscope. ......Fibrous elements, but in small quantity, and principally under the form of fibro-plastic globules, fusiform globules, fibres widened in the middle, and perfect fibres, are also visible."

To the head of epidermic tumours the author refers corns, callosities, and condylomata, and besides a—

> "Species of pure epidermic tumour, of papillary form, occasionally found on various parts of the body, and by no means always traceable to the syphilitic virus as its cause. Tumours of this kind may even acquire considerable bulk and become tolerably vascular; they are susceptible of becoming inflamed, of ulcerating, and of suppurating. Viewed with the naked eye they then appear covered, first, with a layer of variable thickness; when this layer is removed a yellowish liquid is discovered, composed of globules of pus and epidermis, mixed sometimes with sebaceous matter. These tumours are in general of yellow or reddish-yellow colour, elastic, and composed of papilliform lobules, somewhat elongated or united into groups, and exhibiting the cauliflower aspect. The microscope only discloses the elements of epidermis and a few vessels, and in the crusts an admixture of pus and epidermis. In this class we place a good number of tumours of the lower lip, supposed to be cancerous, but which on careful examination are found deficient in the elements of cancer, and composed in reality of hypertrophied papillæ, protruding from the surface of a superficial ulceration, and seated on an indurated basis, resulting from chronic inflammation."

To the fact that such sores as this are frequently mistaken for cancerous ulcers, M. Lebert ascribes the long current belief that carcinoma of the lip is more effectually curable by operation than the same disease in other parts.

A fourth species of epidermic tumour the author calls the fibro-epidermic; none other than warts of different kinds. A fifth species, of very rare occurrence, is formed by the simultaneous development of a great number of papillæ, accompanied with hypertrophy of the subaceous glands, and constituting large cauliflower tumours, covered with a sebaceous or purulent fluid wherever ulceration has taken place. A sixth species of epidermic tumour is found, of a "fibrous stroma, resembling that met with in cancer. But this stroma, instead of containing the juice and the tissue proper to cancer, is filled with a whitish grumous substance, in which microscopical examination discloses nothing but epidermal elements." A seventh species consists of epidermic tumours formed of cutaneous cysts, the investment and contents of which are composed of epidermis.

Ophthalmologists are much interested in the question of the precise nature and mode of origin of certain tumours of the eye, quite as certainly curable by operation as cancerous productions in that organ are beyond the reach of surgical cure. There is no doubt that some such tumours are purely fibrinous in composition, and that they are the reliquiæ of extravasations of blood. M. Lebert gives a description of a little tumour of this sort which was seated in front of the retina, and probably owed its existence to hemorrhage from one of the vessels of the choroid. The tumour was of about the size of a cherry; it was of blackish-red colour internally, a fact showing that it was probably of recent origin. In the midst of the portions thus deeply coloured appeared others of a grayish-yellow tint, having precisely the aspect of coagulated fibrine. Delicately granular fibrine in coagula, "condensed under the form of muscular fasciculi," was alone discoverable under the microscope. Blood-corpuscles, more or less completely deprived of their colouring matter, were also visible.

M. Lebert very correctly raises his voice against the use of the term sarcoma as deficient in precision, and as having been applied, more especially perhaps in its compound form osteosarcoma, to all varieties of morbid growths; but M. Lebert is not the first who has perceived the error, or denounced its followers. That a true and real tumour exists, possessing an active power of growth, and assuming many of the outward characters of cancer, to which the name sarcoma has been applied, has long been known; and the difficulty of distinguishing the two kinds in all cases was made even more evident than it had before been by Müller, who showed, as he believed, that the microscopical constituents of the two growths might be, and were in some cases, perfectly similar, and also discovered that the chemical basis of both was albumen. M. Lebert, descrying the clinical importance of the distinction, engages in an attempt to establish it. He describes these tumours under the name of fibro-plastic tumours, and considers them to be composed of the species of globule of that name, of which we have already given a condensed account. The distinctions will then turn on the characters of the globule; and upon this point we must allow M. Lebert to speak:

"These globules are characterized by having a pale cell-membrane as their wall, and a nucleus with very clearly-defined and black outline under the microscope. The involucrum gives a mean diameter of ·015 of a millimeter, and the nucleus varies between ·0075 and ·01 of a millimeter. The entire globules are either spherical or ovoid; and it is only on superficial examination that they can be confounded with the globules of encephaloid. The latter are much paler; they are generally only found under the form of round or oval nuclei, tolerably isolated, larger than the nuclei of the fibro-plastic globule, and also containing nucleoli larger than those of the globule of sarcoma, which are scarcely to be seen under the microscope, except as small black points. The involucrum of the encephaloid globule, when well developed, is much less regular and commonly flattened, finely punctuated, often infiltrated with fat, either in the state of granules or liquid; whereas fatty elements are rarely met with in fibro-plastic tissue, and when they do exist in it are always present in small quantity. Cancerous globules may certainly be more or less elongated; but the intermediate degrees between the fibre and the cell, which characterize the elements of cellular tissue in progress of formation identical to that of the fibro-plastic tissue, are much less frequent in cancerous substance. *In pointing out the existence of such elements in encephaloid cancer, observers have fallen into the serious error of taking an accidental for an essential element; and the cellular tissue formed out of fibro-plastic globules in cancer (and which, it may be added, does not often occur therein under that form), no more constitutes its characteristic and essential element than the various fatty substances which exist in it much more frequently.*" (vol. ii, p. 133.)

It remains for future observation to decide whether the points of more or less nice distinction referred to by M. Lebert really prevail in the two kinds of cell referred to. We have printed a portion of the above extract in italics, in order to show that the idea of the passage was also struck out by Dr. Walshe. This writer, speaking in the volume to which we have before referred, of the production of fibre in the substance of cancer, observes:

"The spherical cancer-cell being formed, the production of the caudate cell follows as an effect of elongation of opposite points of its circumference. And these caudate cells eventually pass into the state of filaments, and form the elements of fibrous formation. Such is the generally admitted notion. But it appears to me that, as matter of theory, caudate cells must also be produced from spherical non-cancerous cells; inasmuch as fibres, in no important point distinguishable from those of natural cellular tissue, are produced in these growths. And *it is certain that the caudate corpuscles visible within cancerous tumours do exhibit different characters;* though to classify them, and connect one more particularly than another kind with the subsequent fibre is at present beyond my power."

It appears from the admission in this last sentence, that if M. Lebert's statements be proved to be correct, he has advanced a step beyond our countryman.

The other microscopical elements which M. Lebert has met with in these tumours are—2. Mother cells which occasionally reach a twelfth of a millimeter in diameter, and contain eight, ten, twelve, or more nuclei and fibro-plastic globules in their interior. 3. The greater part of the mass is composed of fusiform corpuscles. 4. True fibres are almost always met with in tumours of this kind. 5. In fleshy sarcomata a fair portion of the growth is composed of very small globules, which only present from ·005 to ·0075 of a millimeter in diameter; they are possibly fibro-plastic nuclei.

In the author's description of fibrous tumours we recognize the evidence of minute and diligent study of these products, but there is no novel fact established or brought forward.

The account of enchondromatous and osteoid tumours is borrowed (with acknowledgment) from Müller.

M. Lebert's chapter on Cancer is elaborate and valuable. The chief fact put forth in it of novel character is that the cell of cancer is distinguishable by its properties from all other cells, either of morbid or natural origin. We shall not submit this chapter to analysis, however, as much of our present Number is devoted to the subject of cancer in connexion with Dr. Walshe's recent volume. But we believe that we may interest some of our readers at least by noticing a few points in which we have remarked, in perusing these two works, that the Swiss and English observers differ. Dr. Walshe lays singular stress upon the property possessed by cancer of infiltrating the natural tissues amid which it is developed. This property, indeed, he goes so far as to maintain gives cancer its place apart among GROWTHS properly so called. M. Lebert also makes much of this character of cancerous formations. But the mode of explaining the phenomenon adopted by the two writers is very widely different. The explanations are thus given:

"Cancer," says M. Lebert, "like tubercle, possesses the property of being deposited by infiltration," [tubercle is not a GROWTH, however; so that here there is not any *expressed* difference of opinion from Dr. Walshe,] "because, being often constitutional maladies, they are deposited by a much greater number of capillary vessels, than are, in general, local hypertrophy and the adventitious production of a normal tissue." (vol. ii, p. 246.)

This idea, not very elegantly expressed, certainly appears in contradiction with opinions taught by the author himself in other parts of his work. Thus he regards fibrous and certain other tumours as evidences, in certain cases, of a constitutional state, and yet these tumours do not infiltrate the circumjacent textures. The following explanation of Dr. Walshe, if not destined to be ultimately received, is at least less easily overturned:

"Considered abstractedly, there seems nothing very terrible in this property [of infiltration]. So clearly is this the fact, that it appears to me we must look elsewhere for an explanation of the evils practically connected with infiltration, than to the mere physical phenomenon itself; in a word, we must seek elsewhere some condition, of which the process is but a consequence or involution. But the natural place to look for this condition is *in the tissues themselves*, which undergo infiltration; and in these—in some special morbid change within them—must reside the source and origin of the process. And this view is confirmed by the fact, that there is nothing in the mode of vegetation of cancer itself to explain why it alone, among vegetating new formations, should possess the power of infiltrating the natural tissues."

We do not find in Dr. Walshe's work any distinct attempt made to establish an anatomical diagnosis (if we may so speak) between the cells of encephaloid, scirrhus, and colloid. The continuous enlargement of the cells of colloid, while those of scirrhus appear to retain persistently their primitive dimensions, and the fact that the nucleus of the encephaloid cell is larger than that of the scirrhous cell, are the chief points he draws attention to. M. Lebert aims, as follows, at more precise distinction:

"The globule of fibrous, hard, and *scirrhous* cancer is generally supplied with a membrane of investment of round, ovoid, or irregular shape. The mean diameter varies between ·015 and ·02 of a millimeter; it is finely punctuated round the nucleus. The latter is small, varying between ·0075 and ·01 of a millimeter in diameter, with a strongly marked outline, and exhibiting in its interior granules amorphous particles, and not unfrequently nucleoli.

"In the *encephaloid* globule the involucrum is regular or irregular, and of a mean diameter of from ·02 to ·03 of a millimeter. The nucleus is spherical, very often elliptical, pale, shaded at the periphery, and containing from one to three very distinct nucleoli. These nucleoli in general are more abundant than the complete cells." (vol. ii, p. 258.)

But whatever precision of information may be deduced from this comparison undergoes very serious modification from a statement which is added, that "all intermediate forms between these two types are observed." The question is briefly this: Can the naked-eye characters (in other words, the species) of a cancerous tumour be always decided from microscopical inspection of its *elementary cell?* We are certainly disposed to believe it cannot; but we admit our inability to decide the question.

We notice a statement here by the author, that he has sometimes found a "fibroid rete," containing cancerous cells within it, and "perfectly resembling coagulated fibrine." This is an important observation, which has also been made by Dr. Walshe, who (op. cit. p. 58) draws an inference of some consequence from it, as to the production of blastema in connexion with extravasated blood.

M. Lebert conceives that the yellow discoloration often witnessed in cancerous tumours (particularly in encephaloid of the testicle) is due to a particular species of colouring matter, for which he proposes the name of *xanthosis.* Attentive examination has, he says, proved that this material is not altered colouring matter of the blood, but that it is constituted by a particular species of fat or oil.

There is little to interest in the account of cancer given by M. Lebert, except his purely anatomical and microscopical details. Some few vague observations on the influence of age and sex on the disease are really amusing from their simplicity. A distinct paragraph, for example, is devoted to the important announcement: "In general, I *think* that cancer is more frequent in the female than in the male." Think! And why should not M. Lebert think? A man of his powers is doubtless much more likely to come to accurate conclusions on such a question by thinking, than by consulting the statistical tables supplied by the government returns in this country, and the numerous valuable numerical communications of various foreign writers on the subject.

Two detached essays, one on the formation of callus, the other on the minute anatomy and treatment of true porrigo favosa, close the volumes. There is no new mode of treatment of the latter affection suggested, except that of picking out with the point of a needle the little vegetables which constitute its essential element. At least this mode of treatment is new to us, though possibly it may have already been put in practice by others. M. Lebert speaks well of the iodide of sulphur; but we *know* that it will repeatedly fail, and doubt its ever having cured the disease. The whole account of this disease is, however, very excellently done, and will well repay perusal.

Had the work we have now passed in review been put forward as a mere compilation, accompanied with illustrative observations—had it been less crowded with the author's self-gratulations—had it not started with an announcement that M. Lebert commenced his medical studies with a sort of contemptuous pity for the false philosophy prevailing among his predecessors—had justice been done to those predecessors—had any shadow of improvement been made upon the philosophy of those predecessors—had all this been the case, M. Lebert's claims to the courteous reception of his brethren would have been far different from what they now are. While we do not see that he has added anything of real consequence, either in fact or doctrine, to what was already made out, he has, however, produced a work of laborious and, in some respects, useful character. Yet, on the other hand, his accuracy of description may often be doubted. We do not, for example, believe in his tuberculous corpuscle, nor in his "pyoid" corpuscle. Nor can we avoid commenting unfavorably on the figures in his plates. Many of the objects are new to us—of these we therefore say nothing; but wherever the thing represented is one with which we are familiar, the wretchedness of the delineation has deeply impressed us. In fact, we can affirm that it requires much familiarity with the objects to guess what they mean; and, in a word, the figures are apparently made from the *descriptions* of the objects, and not from those *objects themselves.*

---

